# Cytotoxic Tricycloalternarene Compounds from Endophyte *Alternaria* sp. W-1 Associated with *Laminaria japonica*

**DOI:** 10.3390/md16110402

**Published:** 2018-10-23

**Authors:** Li Shen, Shu-Juan Tian, Hui-Liang Song, Xi Chen, Hao Guo, Dan Wan, Yu-Rou Wang, Feng-Wu Wang, Li-Jun Liu

**Affiliations:** 1 Institute of Translational Medicine, Medical College, Yangzhou University, Yangzhou 225001, China; shenli@yzu.edu.cn (L.S.); 18362825104@163.com (H.-L.S.); cx0811chenxi@163.com (X.C.); 18752789443@163.com (H.G.); 18252717215@163.com (D.W.); xiaorouw@gmail.com (Y.-R.W.); 2 Jiangsu Key Laboratory of Integrated Traditional Chinese and Western Medicine for Prevention and Treatment of Senile Diseases, Yangzhou University, Yangzhou 225001, China; 3 Jiangsu Co-Innovation Center for Prevention and Control of Important Animal Infectious Diseases and Zoonoses, Yangzhou 225009, China; 4 College of Food Science and Engineering, Qingdao Agricultural University, Qingdao 266109, China; tianshujuan1992@163.com; 5 Jiangsu Co-Innovation Center for Modern Production Technology of Grain Crops/Jiangsu Key Laboratory of Crop Genetics and Physiology, Yangzhou University, Yangzhou 225009, China

**Keywords:** *Laminaria japonica*, endophyte, *Alternaria* sp., tricycloalternarene compound, cytotoxicity

## Abstract

The chemical investigation of the culture filtrate of endophyte *Alternaria* sp. W-1 associated with *Laminaria japonica* provided a new tricycloalternarene compound, 2H-(2*E*)-tricycloalternarene 12a (**1**), together with five known analogs: (2*E*)-tricycloalternarene 12a (**2**), tricycloalternarene 3a (**3**), tricycloalternarene F (**4**), 15-hydroxyl tricycloalternarene 5b (**5**), and ACTG-Toxin D (**6**). In vitro cytotoxicity against the human hepatocellular carcinoma cell line SMMC-7721 and the human gastric carcinoma cell line SGC-7901 was evaluated by the MTT method. Compounds **1**, **3**, and **4** inhibited the growth of SMMC-7721 cells with IC_50_ values of 49.7 ± 1.1, 45.8 ± 4.6, and 80.3 ± 3.8 μg/mL, respectively, while the IC_50_ value of the positive control cisplatin was 6.5 ± 0.5 μg/mL. Compounds **3** and **6** also showed moderate anti-proliferation activity against SGC-7901 cells with IC_50_ values of 53.2 ± 2.9 and 35.1 ± 0.8 μg/mL, respectively, while the IC_50_ value of cisplatin was 4.5 ± 0.6 μg/mL. Further studies revealed that the in vitro anticancer activity of compound **3** to SMMC-7721 cells was related to G1 phase cell cycle arrest and cell apoptosis, and the induced apoptosis was involved in both the mitochondrial pathway and the death receptor pathway. This is the first report on the anticancer mechanism of tricycloalternarene compounds.

## 1. Introduction

Marine microorganisms survive under extreme conditions, including the absence of light, low level of oxygen, and intensely high pressure, which often leads to modifications of primary and secondary metabolic pathways and an increased likelihood of producing natural products with unique structures and/or significant activities that differ from those produced by terrestrial microorganisms. Most marine microorganisms are in symbiotic, parasitic, and epiphytic relationships with marine plants (algae, intertidal plants, and mangroves) and marine animals (cnidarias, arthropods, mollusks, tunicates, and echinoderms) [1,2]. The seabed sediment, mangrove plants, sponges, and algae with relatively rich nutrition are the main habitats or hosts for marine microorganisms that produce new and/or bioactive compounds [3].

**Figure 1:**
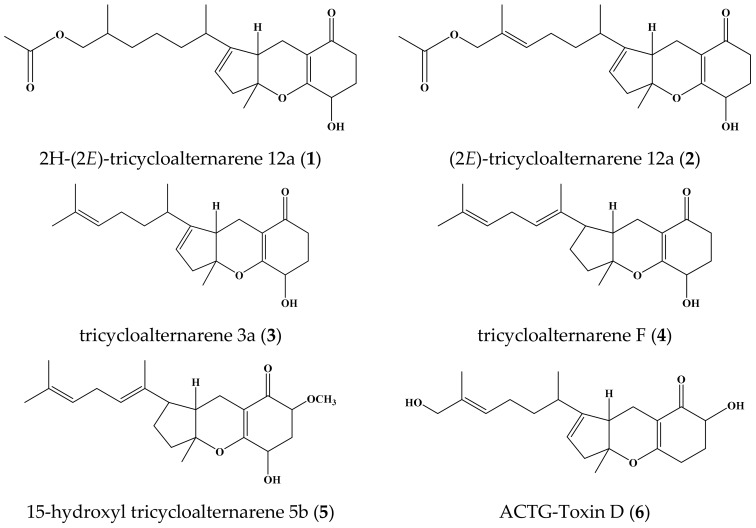
Chemical structures of compounds **1**–**6**.

*Laminaria japonica*, also called kelp in Chinese medicines, is a kind of large marine plant. In our continuous research on endophytes associated with marine plants, the endophytic fungus *Alternaria* sp. W-1 was isolated from *L. japonica* in Weihai, China. *Alternaria* is a cosmopolitan fungal genus widely distributed in soil and organic matter. It includes saprophytic, endophytic, and pathogenic species. *Alternaria* sp. was reported to produce various bioactive natural products mainly including nitrogen-containing metabolites, steroids, terpenoids, pyranones, quinones, and phenolics [4]. Guided by the ^1^H-NMR spectrum and thin-layer chromatography, the chemical investigation of an ethyl acetate extract of the culture filtrate of *Alternaria* sp. W-1 was carried out, and a new tricycloalternarene compound, 2H-(2*E*)-tricycloalternarene 12a (**1**) was obtained. In addition, five known analogs—(2*E*)-tricycloalternarene 12a (**2**) [5], tricycloalternarene 3a (**3**) [6], tricycloalternarene F (**4**) [7], 15-hydroxyl tricycloalternarene 5b (**5**) [8], and ACTG-Toxin D (**6**) [9] (Figure 1)—were isolated from the liquid culture of *Alternaria* sp. W-1.

Tricycloalternarenes, related to ACTG-toxins, are a kind of meroterpene found in *Alternaria* sp. [5,10,11], *Ulocladium* sp. [7], *Guignardia* sp. [12], *Paradendryphiella* sp. [13], etc. They were first known as host-selective toxins from *Alternaria citri*, which caused brown spot disease of the Dancy tangerine (*Citrus reticulata*) and other mandarin cultivars [14,15]. Tricycloalternarene compounds have various bioactivities, including NF-κB inhibitory activity [16], anti-inflammatory properties [17], cytotoxicity against tumor cell lines [7,18], antimicrobial activity [19], inhibition activity against the Bacille Calmette-Guerin strain [7], and the ability to inhibit the growth of marine phytoplankton [5,10,20]. As a class of potential lead compounds, tricycloalternarene compounds are worthy of further research concerning their structure and pharmacological activity. Here, the in vitro cytotoxicity of six tricycloalternarene compounds (**1**–**6**) are reported, and the details of their isolation and structure are elucidated.

## 2. Results and Discussion

### 2.1. Structure Elucidation

**Figure 2:**
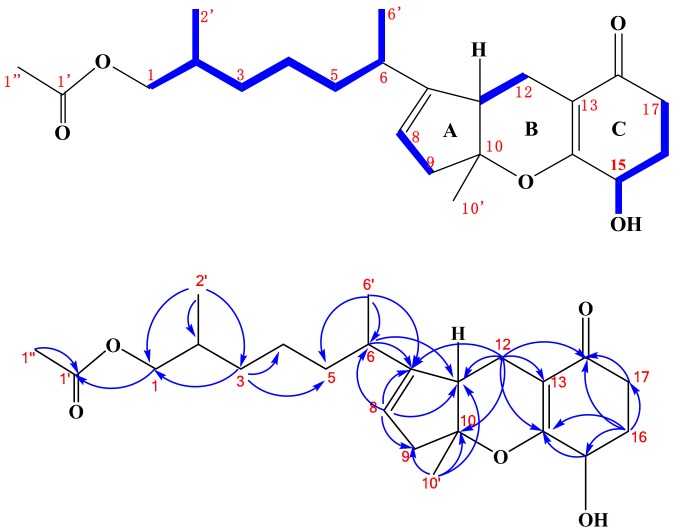
Key ^1^H-^1^H COSY (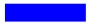) and HMBC (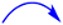) correlations of compound **1**.

Compound **1** was isolated as a colorless oily solid and its molecular formula was deduced to be C_23_H_34_O_5_ according to the HR-ESI-MS spectrum, possessing seven degrees of unsaturation. The ^1^H-NMR spectrum of **1** showed the presence of two methyl singlets at δ_H-10′_ 1.4 and δ_H-1″_ 2.0 ppm, two methyl doublets at δ_H-2′_ 0.81 and δ_H-6′_ 0.90 ppm, one olefinic hydrogen *at* δ_H-8_ 5.31 ppm, one hydroxyl hydrogen at δ_15-OH_ 5.38 ppm, one oxy methine hydrogen at δ_H-15_ 4.01 ppm, and a pair of coupled oxy methylene hydrogens at δ_H-1ab_ 3.83 and 3.75 ppm (Figure S1). The ^13^C-NMR spectrum of **1** exhibited 23 resonances, classified into two carbonyl carbons, four olefinic carbons, four methyl carbons, eight methylene carbons, four methine carbons, and one quaternary carbon (Figure S2). There must be three rings in the structure of **1**, deduced by its NMR data and degrees of unsaturation. The ^1^H- and ^13^C-NMR spectra of **1** displayed the characteristics of the tricycloalternarene compound and were very similar to those of (2*E*)-tricycloalternarene 12a (**2**) (molecular formula C_23_H_32_O_5_, two hydrogens less than **1**) [5]. This indicated that compound **1** might be a hydrogenated derivative of (2*E*)-tricycloalternarene 12a (**2**). A detailed NMR comparison revealed that the ^1^H- and ^13^C-NMR data of **1** from H-5 to H-17 and from C-5 to C-18 were very similar to those of (2*E*)-tricycloalternarene 12a (**2**). However, there were some obvious NMR differences between **1** and **2**. Compared with (2*E*)-tricycloalternarene 12a (**2**) [5], the absence of an olefinic hydrogen at δ_H-3_ 5.38 ppm and a methyl doublet at δ_H-2′_ 0.81 ppm instead of a methyl singlet at δ_H-1″_ 2.0 ppm were observed in the ^1^H-NMR spectrum of **1**. In addition, two olefinic carbons at δ 130.2 and 129.7 ppm were missing in the ^13^C NMR spectrum of **1**, supporting the notion that the double bond between C-2 and C-3 in **2** was hydrogenated to transform into **1**. Carbon signals at δ_C-15_ 64.6 and δ_C-17_ 32.2 ppm in **1** differed from those in tricycloalternarene D and tricycloalternarene E [11]. Furthermore, the ^1^H-^1^H COSY correlation between δ_H-15_ 4.01 and δ_15-OH_ 5.38 ppm (Figure S5) and the HMBC correlation from δ_H-15_ 4.01 to δ_C-14_ 170.8 ppm (Figure S4) also proved that the secondary hydroxyl group at δ 5.38 ppm in ring C was placed at C-15. The key ^1^H-^1^H COSY and HMBC correlations, shown in Figure 2, confirmed the planar structure of **1**.

Then, the relative configuration of **1** was determined based on the following information. The observed NOE correlation between δ_H-10′_ 1.40 and δ_H-11_ 2.76 ppm (Figure S6) suggested that rings A and B in **1** were *cis*-fused [21]. The small vicinal coupling constant of H-15 (t, *J* = 4.8 Hz) in **1** implied an equatorial orientation of H-15 [21], and C-15 in **1** was deduced as an *S*-configuration attributed to the same circular dichroism (CD) spectrum as **2** (Figure S7) [5]. However, the absolute configurations at C-2, C-6, C-10, and C-11 could not be determined due to insufficient data. Finally, compound **1** was determined to be 2H-(2*E*)-tricycloalternarene 12a and its ^1^H- and ^13^C-NMR data were unambiguously assigned by a series of 2D NMR experiments (HSQC, HMBC, ^1^H-^1^H COSY, and NOESY, Figures S3–S6), as shown in Table 1.

### 2.2. Cytotoxicity of Tricycloalternarene Compounds

**Figure 3:**
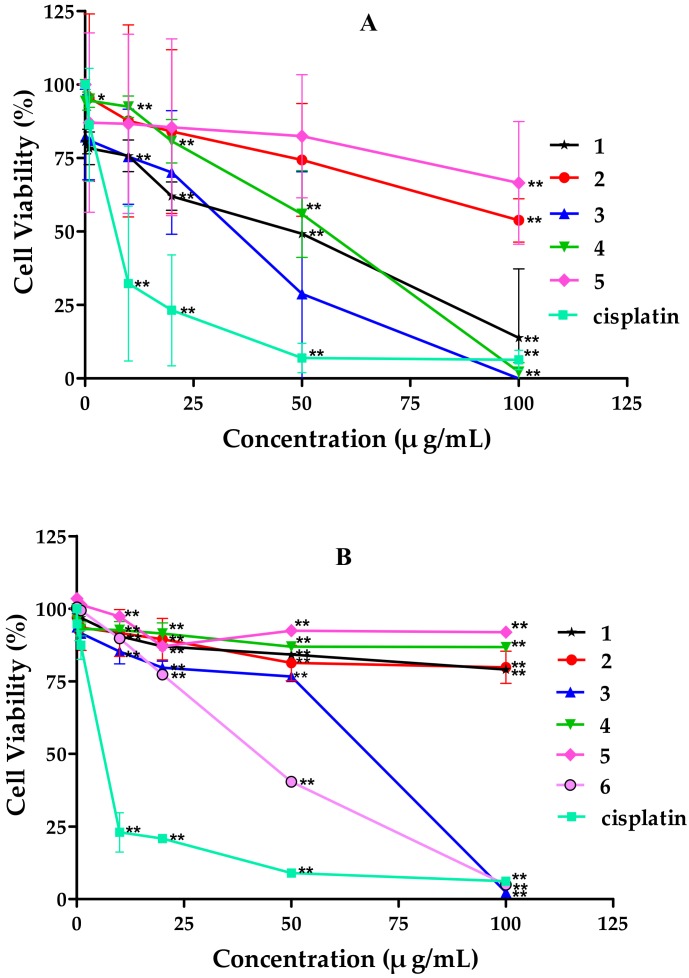
Effects of tricycloalternarene compounds on the proliferation of: SMMC-7721 cells (**A**); and SGC-7901 cells (**B**). The cell proliferation rate was examined after treatment with compounds **1**–**6** (0.1, 1, 10, 20, and 50 μg/mL) for 48 h. Compared with the negative control (NC), * *p* < 0.05, ** *p* < 0.01.

The in vitro cytotoxicity of six tricycloalternarene compounds to the human hepatocarcinoma cell line SMMC-7721 and the human gastric carcinoma cell line SGC-7901 were evaluated by the MTT method. Compounds **1**, **3**, and **4** inhibited the proliferation of SMMC-7721 cells in a dose-dependent manner with IC_50_ values of 49.7 ± 1.1, 45.8 ± 4.6, and 80.3 ± 3.8 μg/mL, respectively. Meanwhile, the positive control cisplatin showed anti-proliferation activity against SMMC-7721 cells with an IC_50_ value of 6.5 ± 0.5 μg/mL (Figure 3). Compounds **3** and **6** also suppressed the growth of SGC-7901 cells moderately in a dose-dependent manner, with IC_50_ values of 53.2 ± 2.9 and 35.1 ± 0.8 μg/mL, respectively, while cisplatin exhibited cytotoxic activity against SMMC-7721 cells with an IC_50_ value of 4.5 ± 0.6 μg/mL (Figure 3).

### 2.3. Tricycloalternarene 3a (**3**) Induced G1 Phase Cell Cycle Arrest in SMMC-7721 Cells

**Figure 4:**
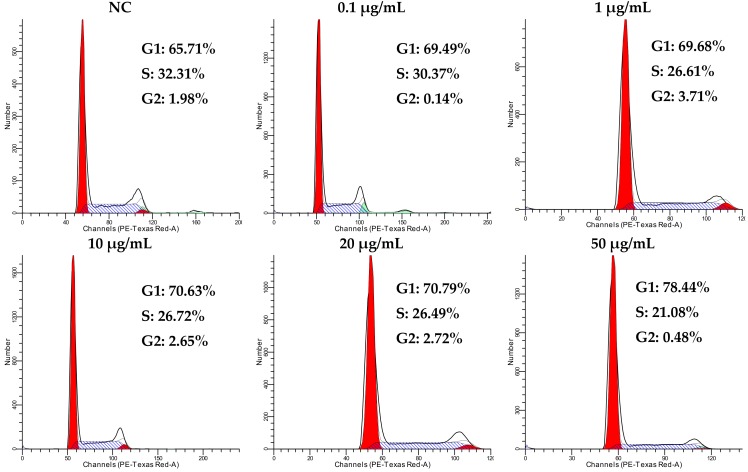
Influence of tricycloalternarene 3a (**3**) on the cell cycle of SMMC-7721 cells. A cell cycle analysis of SMMC-7721 cells was carried out after 48 h of treatment with tricycloalternarene 3a (0.1, 1, 10, 20, and 50 μg/mL).

Cell proliferation is precisely regulated by the cell cycle; however, the dysregulation of the cell cycle is an intrinsic factor of tumor occurrence. Tricycloalternarene 3a (**3**) inhibited the growth of SMMC-7721 cells moderately in an MTT assay. To discover whether the anti-proliferation activity of compound **3** against SMMC-7721 cells was associated with cell cycle arrest, a cell cycle analysis of SMMC-7721 cells treated with tricycloalternarene 3a for 48 h was carried out. The results show that, compared to the negative control (NC), the percentages of treated SMMC-7721 cells in G1 phase were increased from 65.71% to 69.49%, 69.68%, 70.63%, 70.79%, and 78.44% (Figure 4), which implied that compound **3** could induce cell cycle arrest in SMMC-7721 cells in G1 phase.

Cell cycle progression is mainly co-regulated by cyclin, cyclin-dependent kinase (CDK), and cyclin-dependent kinase inhibitor (CDKI). CDKI can be divided into two families: inhibitors of cyclin-dependent kinase 4 (INK4) with specific inhibition to cyclin D-CDK4/CDK6 (including p15, pl6, p18, and p19) and cyclin-dependent kinases-interacting protein (CIP)/kinase-interacting protein (KIP) with non-specific effects (including p21, p27, and p57) [22]. As a non-specific CDKI, p27 (also known as KIP1) mainly exerts a negative effect on the cell cycle by inhibiting the cyclin-CDK complexes of G1 phase, such as cyclinE-CDK2 and cyclinD-CDK2. cyclinE-CDK2 and cyclinD-CDK2 are key enzymes that promote cells from G1 into S phase in the cell cycle—p27 could inhibit these two G1 kinase complexes to delay the progression of G1 phase in the cell cycle [23,24].

**Figure 5:**
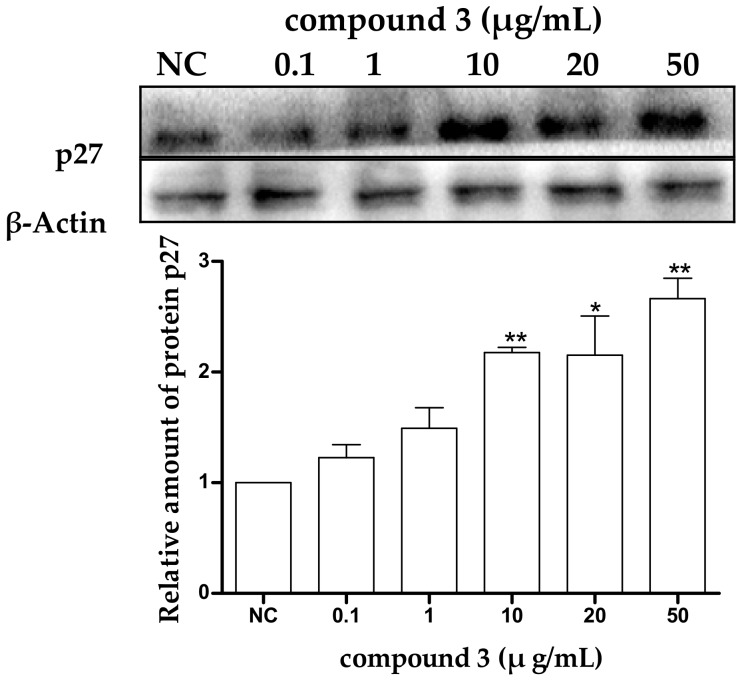
Effect of tricycloalternarene 3a (**3**) on the expression of protein p27 in SMMC-7721 cells. SMMC-7721 cells were treated with tricycloalternarene 3a (0.1, 1, 10, 20, and 50 μg/mL) for 48 h. Compared with negative control (NC), * *p* < 0.05, ** *p* < 0.01.

To confirm the effect of protein p27 in tricycloalternarene 3a-induced G1 phase cell cycle arrest in SMMC-7721 cells, the expression level of p27 in treated SMMC-7721 cells was examined by Western blot analysis. Untreated cells were used as a negative control (NC) and β-actin was used as an internal reference. It could be seen that expression level of p27 in treated cells was increased obviously (Figure 5), which meant that p27 might participate in the induced G1 phase cell cycle arrest in SMMC-7721 cells. Protein p27 is already considered as a potential tumor suppressor protein, and the abnormal expression of the p27 gene is closely related with the occurrence and development of multiple malignant tumors [22,25]. Many extracellular anti-proliferation signals have been reported to induce the expression of p27 and prevent the cell cycle from proceeding from G1 phase to S phase, thus inhibiting cell proliferation. The upregulation of p27 in the present study demonstrated that the anti-proliferation effect of tricycloalternarene 3a against SMMC-7721 cells might be achieved by p27-mediated G1 phase cell cycle arrest to some extent.

### 2.4. Tricycloalternarene 3a (**3**) Induced Apoptosis in SMMC-7721 Cells

**Figure 6:**
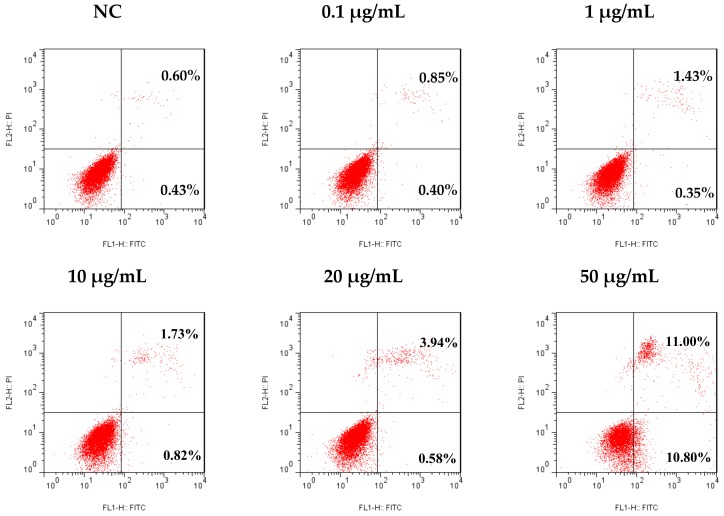
Effect of tricycloalternarene 3a (**3**) on the apoptosis rate of SMMC-7721 cells. SMMC-7721 cells were treated with tricycloalternarene 3a (0.1, 1, 10, 20, and 50 μg/mL) for 48 h.

There is a balance between apoptosis and cell proliferation. As a type I programmed cell death (PCD), apoptosis plays a negative regulatory role in tumor formation and development. It had been found that the pathogenesis of many tumors is closely related to the obstruction of apoptosis [26,27]. In the present study, an Annexin V-FITC/PI dual stain assay was used to analyze the apoptosis rate of SMMC-7721 cells after 48 h of treatment with different concentrations of tricycloalternarene 3a (**3**), to determine whether the anti-proliferation effect of compound **3** against SMMC-7721 cells was associated with induced apoptosis. The result showed that compound **3** induced apoptosis in SMMC-7721 cells in a dose-dependent manner. Compared with the negative control (NC), the apoptosis rate of treated cells was increased from 1.03% to 1.25%, 1.78%, 2.55%, 4.52%, and 21.8%, respectively (including early apoptosis and late apoptosis, Figure 6), indicating that tricycloalternarene 3a could enhance apoptosis in SMMC-7721 cells and inhibit SMMC-7721 cell proliferation via this induced apoptosis. 

Cell apoptosis is an autonomic ordered programmed cell death strictly regulated by multiple genes, such as those of the Bcl-2 (B cell lymphoma-2) family, caspase family, as well as oncogenes and anti-oncogenes. The Bcl-2 protein family, the key regulator of apoptosis, is comprised of anti-apoptotic members (such as Bcl-2, Bcl-xL, Bcl-W, Bfl-1, and Mcl-1) and pro-apoptotic members (such as Bax, Bak, Bad, Bcl-XS, Bid, Bik, Bim, and Hrk) [28]. The *bcl*-2 proto-oncogene is the major gene to inhibit cell apoptosis, and the overexpression of *bcl-2* gene has been observed in many types of tumor cells and tissues [29]. Recently, the targeting of anti-apoptotic subfamily of proteins has become a new method to improve treatment outcomes for cancer patients. The plant-derived natural product Gossypol (AT-101), directed at inhibiting Bcl-2, has been in clinical trials with the aim of curing recurrent extensive stage small cell lung cancer [30,31].

**Figure 7:**
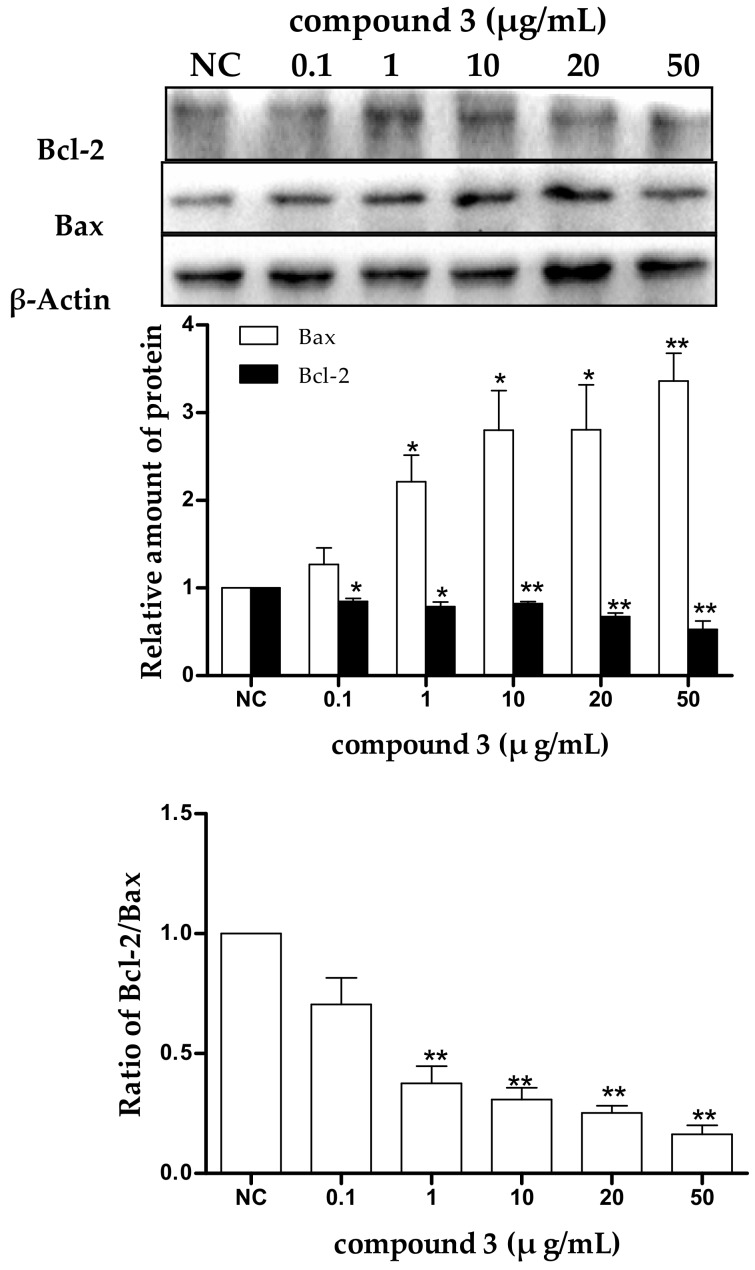
The effect of tricycloalternarene 3a (**3**) on the expression of protein Bcl-2 and Bax in SMMC-7721 cells. SMMC-7721 cells were treated with tricycloalternarene 3a (0.1, 1, 10, 20, and 50 μg/mL) for 48 h. Compared with negative control (NC), * *p* < 0.05, ** *p* < 0.01.

To determine whether the tricycloalternarene 3a (**3**)-induced apoptosis in SMMC-7721 cells is associated with Bcl-2 family proteins, the expression levels of the anti-apoptotic protein Bcl-2 and the pro-apoptotic protein Bax in treated SMMC-7721 cells were examined after 48 h of treatment. As shown in Figure 7, in treated cells, the downregulation of Bcl-2 and the upregulation of Bax were observed in a dose-dependent manner and the ratio of Bcl-2/Bax was also decreased obviously with dose-dependence. The ratio between pro- and anti-apoptotic molecules helps determine the susceptibility of cells to a death signal to some extent [32]. Bcl-2 family proteins were found to form homo- and/or heterodimers to regulate cell apoptosis. The reduced ratio of Bcl-2/Bax tended to induce Bax translocation to the mitochondria to form homodimers, resulting in the release of cytochrome c from the mitochondria and the reduction in the mitochondrial membrane potential, which in turn led to cell apoptosis through the mitochondrial pathway. Therefore, it could be speculated that tricycloalternarene 3a (**3**) decreased the ratio of Bcl-2/Bax in SMMC-7721 cells, leading to cell apoptosis via the mitochondrial pathway.

### 2.5. Tricycloalternarene 3a-Induced Apoptosis of SMMC-7721 Cells Involved in Both the Death Receptor Pathway and the Mitochondrial Pathway

**Figure 8:**
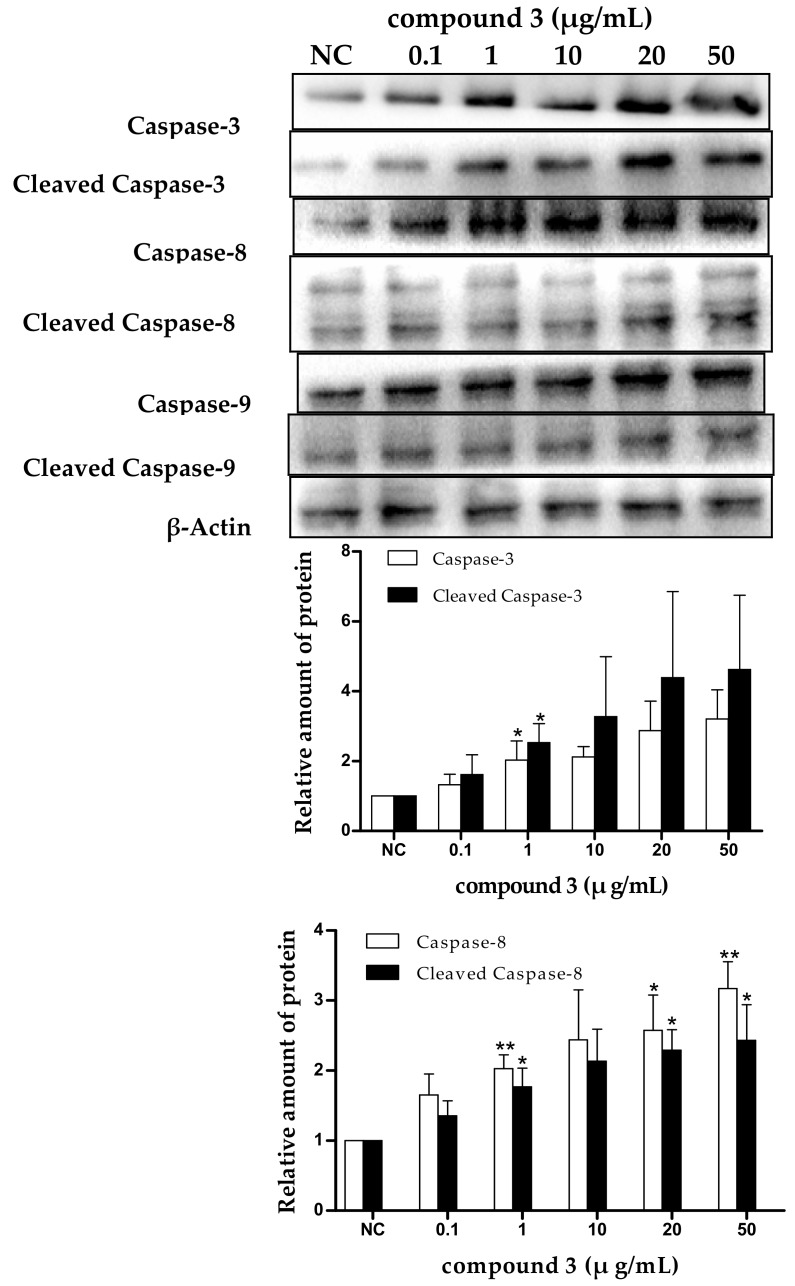

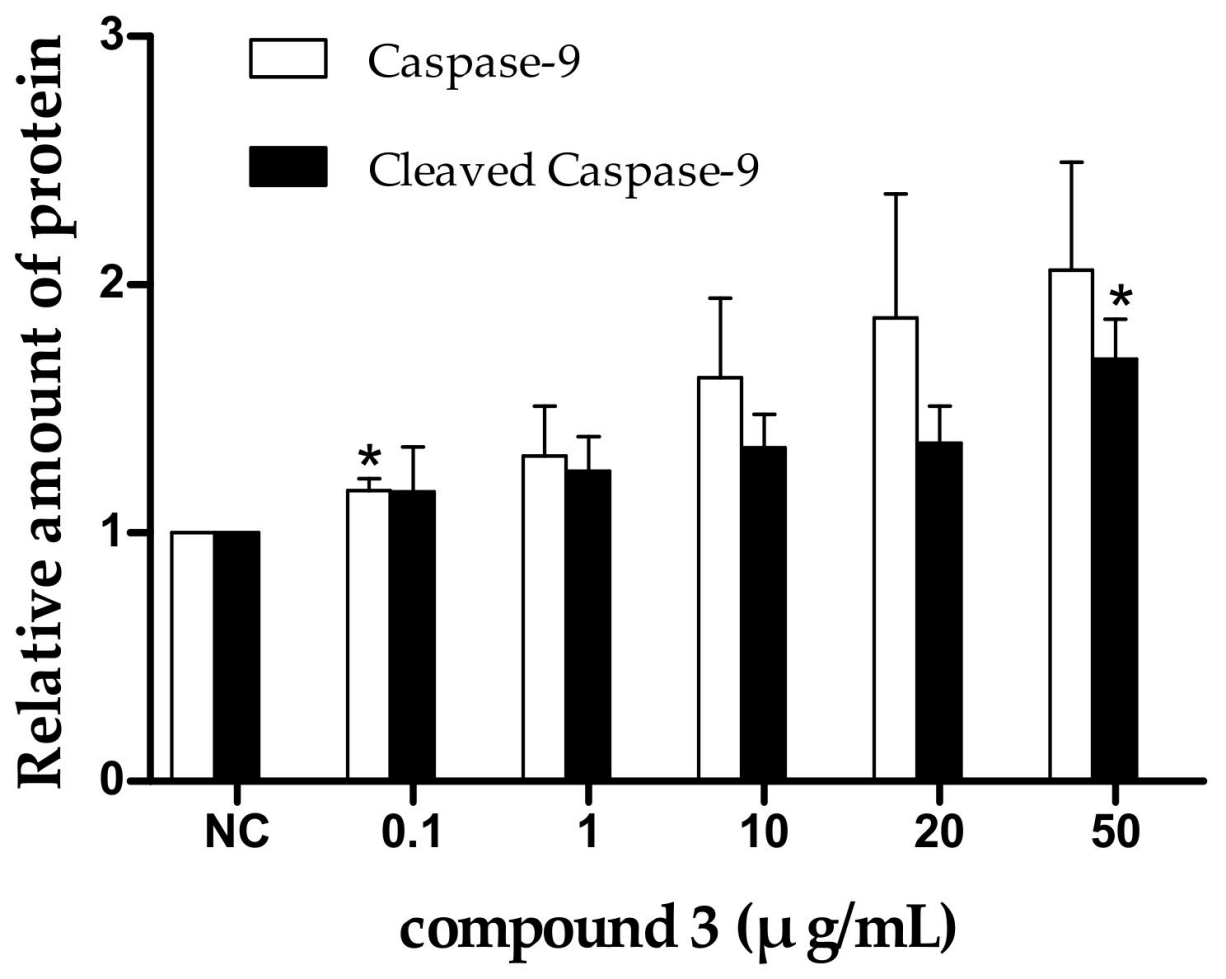
The effect of tricycloalternarene 3a (**3**) on the expression of caspase-3, -8, and -9 and cleaved caspase-3, -8, and -9 in SMMC-7721 cells. SMMC-7721 cells were treated with tricycloalternarene 3a (0.1, 1, 10, 20, and 50 μg/mL) for 48 h. Compared with negative control (NC), * *p* < 0.05, ** *p* < 0.01.

Apoptosis is often an energy-dependent process, which involves the activation of a group of cysteine proteases (i.e., caspases) and a complex cascade of events that link the initiating stimuli to the final demise of the cell [33]. Caspases are family effector proteins that play an important role in apoptosis. They usually activate or deactivate the substrate proteins by protein hydrolysis to regulate apoptosis. To date, 10 major caspases have been identified and broadly categorized into initiators (caspase-2, -8, -9, and -10), effectors or executioners (caspase-3, -6, and -7), and inflammatory caspases (caspase-1, -4, and -5) [33]. Among these, caspase-3 is one of the most important executors of cell apoptosis, and the activation of caspase-3 in apoptosis seems to be an irreversible step towards cell death [34]. To clarify the roles of caspases in tricycloalternarene 3a-induced apoptosis, the expression levels of caspase-3, -8, and -9 and cleaved caspase-3, -8, and -9 were detected by Western blot. As shown in Figure 8, the expression levels of caspase-3, -8, and -9 and cleaved caspase-3, -8, and -9 were all improved in a dose-dependent manner, which verified that tricycloalternarene 3a-induced apoptosis in SMMC-7721 cells was dependent on caspases activation. 

There are two main apoptotic pathways: the extrinsic-death receptor pathway (i.e., the Fas/Fas-L pathway) initiated by caspase-8 and the intrinsic-mitochondrial pathway initiated by caspase-9. Both pathways finally activate caspase-3, which leads to irreversible apoptosis in cells [35]. In the present study, the upregulation of caspase-3 and -8 and cleaved caspase-3 and -8 suggested that the extrinsic-death receptor pathway in tricycloalternarene 3a-treated SMMC-7721 cells was activated. Pro-caspase-8 was activated and active caspase-8 was cleaved followed by the activation of executioner caspase-3, directly leading to nuclear fragmentation and ultimately cell death. The upregulation of caspase-9 and cleaved caspase-9 implied that the intrinsic-mitochondrial pathway in tricycloalternarene 3a-treated SMMC-7721 cells was activated as well. Pro-caspase-9 was activated by non-receptor-mediated stimuli and active caspase-9 was cleaved followed by the activation of executioner caspase-3. Therefore, tricycloalternarene 3a (**3**)-induced apoptosis in SMMC-7721 cells showed caspase-dependence and was involved in both the death receptor pathway and the mitochondrial pathway. 

Cancer, a complex genetic disease resulting from the mutation of oncogenes or tumor suppressor genes, is always regulated by signal pathways [28]. The PI3K/Akt signaling pathway is an important intracellular signal transduction pathway which plays critical roles in cell apoptosis and survival by affecting the activity of downstream effector molecules. The activity of the PI3K/Akt pathway is often abnormally increased in tumor cells. Akt (i.e., protein kinase B, PKB) is a very important downstream target protein of PI3K. Akt could be activated by PI3K through site-specific phosphorylation at Thr308 and Ser473, subsequently resulting in the activation or inactivation of downstream target protein Bad, caspase-9, NF-κB, p21^Cip1^, and p27^Kip1^ to regulate the proliferation, differentiation, apoptosis, and migration of tumor cells [36,37,38,39,40]. The PI3K/Akt signal pathway is closely associated with the development and progression of human tumors; therefore, PI3K and Akt have become potential targets for tumor therapy and a specific inhibitor of the PI3K/Akt signaling pathway would have the potential to become an effective anticancer drug. In the present experiment, tricycloalternarene 3a (**3**) was found to increase the expression levels of p27, Bax, caspase-3, -8, and -9 and cleaved caspase-3, -8, and -9, as well as reduce the expression level of bcl-2. This suggests that mechanisms that PI3K/Akt signaling pathway participated in the tricycloalternarene 3a-mediated anti-proliferation and induced-apoptosis in SMMC-7721 are worthy of further investigation.

## 3. Materials and Methods

### 3.1. General Experimental Procedures

NMR spectra were acquired on an AVANCE 600 NMR spectrometer using solvent signal and tetramethylsilane (TMS) as internal standards. HR-ESI mass spectrum was taken on an UHR-TOF maXis MS instrument. The UV and CD spectra were recorded on a Jasco J-810 circular dichroism spectrometer. HPLC was performed on an Agilent 1260 and a Hitachi Primaide. Flash chromatography was carried out on a Biotage Isolera one. Cells were incubated using a Thermo Series CO_2_ incubator. The absorbance of 96-well plates was measured on a Bio-Tek ELX800UV. Percentages of apoptosis cells were recorded using BD FACSCalibur Flow Cytometry. Western blot analysis was conducted using a Bio-Rad JY-ZY5 Western blot system and a CB FluorChemE automatic gel imaging analysis system.

Human tumor cell lines SMMC-7721 and SGC-7901 were supplied by the American Type Culture Collection, Rockefeller, MD, USA and the Cell Bank of Shanghai, Life Science Research Institute Shanghai, China, respectively. Silica gel (200–300 mesh) for column chromatography was produced by Qingdao Marine Chemical Company, Qingdao, China. Silica gel 60 F_254_ Silica Aluminum sheet (20 × 20 cm) was purchased from H&E Co., Ltd., Beijing, China. Sephadex LH-20 and ODS-A (50 μm) were provided by Pharmacia Biotech, Uppsala, Sweden and YMC CO., LTD., Kyoto, Japan, respectively. HPLC-grade methanol was provided by Tedia Company, Inc., Fairfield, OH, USA. CDCl_3_ and DMSO-*d*_6_ were purchased from Aldrich, St. Louis, MO, USA and Cambridge Isotope Laboratories, Cambridge, MA, USA, respectively. RPMI 1640 medium and Penicillin-Streptomycin mixed liquid were purchased from Hyclone Laboratories Inc., Logan, UT, USA. Fetal bovine serum and 0.25% trypsase were bought from Gibco Co., Grand Island, NY, USA and 3-(4,5-dimethyl-2-thiazolyl)-2,5-diphenyl-2-H-tetrazolium bromide (MTT) was bought from Sigma-Aldrich Co., LLC., St. Louis, MO, USA. Super ECL Plus ultra-sensitive liquid was supplied by Beijing Applygen Technologies Inc., Beijing, China. The Annexin V-FITC apoptosis detection kit was a product of Invitrogen Corporation, Carlsbad, CA, USA. The SDS-PAGE Gel Preparation Kit was purchased from Shanghai Beyotime Biotechnology, Shanghai, China. Propidium Iodide (PI) and the BCA Protein Quantitation Kit were bought from Jiangsu Keygen Biotech Corp., Ltd., Jiangsu, China. The whole protein extraction kit was supplied by Beijing Solarbio Science and Technology Co., Ltd., Beijing, China. Protein Marker was produced by Fermentas International Inc., Burlington, ON, Canada. Monoclonal antibodies against human Bcl-2, Bax; caspase-3, -8, and -9; cleaved-caspase-3, -8, and -9; p27; and β-actin were all purchased from Cell Signaling Technology, Inc., Boston, MA, USA. Horseradish peroxidase-labeled anti-rabbit and anti-mouse IgG secondary antibodies were purchased from Cell Signaling Technology Inc., Boston, MA, USA and Wuhan Boster Biological Technology, Ltd., Wuhan, China, respectively. Cisplatin was provided by Jiangsu Hansoh Pharmaceutical, Jiangsu, China. The 96-well plates were supplied by Corning Inc., Corning, NY, USA. The PVDF membrane was bought from Millipore Corporation, Billerica, MA, USA. Hypersil ODS2 column (5 μm, 4.6 × 250 mm) and Sinochrom ODS-AP column (5 μm, 4.6 × 200 mm) were produced by Dalian Elite Analytical Instruments Co., Ltd., Dalian, China.

### 3.2. Strain

Endophytic fungus W-1 was isolated from wild and fresh *Laminaria japonica* collected from the Weihai sea area, China and identified as *Alternaria* sp. through morphological characterization and by comparing the 18S rDNA sequence (1401 bp) with those of standard records. The sequences of *Alternaria* sp. W-1 were deposited in GenBank (accession No. MF184928). The live culture of *Alternaria* sp. W-1 was kept at the China General Microbiological Culture Collection Center (CGMCC No. 15181).

### 3.3. Extraction and Isolation

*Alternaria* sp. W-1 was cultured through liquid-state fermentation. Briefly, the strain was inoculated into potato dextrose (PD) medium and cultivated in a rotary shaker at 28 °C and 160 r/min for 3 days. Then, the culture liquid was seeded into seawater Czapek–Dox medium with 10% inoculation and left to cultivate under the same conditions for 10 days.

The fermentation broth was extracted with ethyl acetate (10 L × 3) at room temperature to afford 20 g crude extract after in vacuo evaporation of the solvent. The crude extract was fractionated over a silica gel column eluted with a gradient of CH_2_Cl_2_-CH_3_OH (*v*/*v* 100:0~0:100) to give 10 fractions. Fr.2 (2.25 g) was subjected to further column chromatography over silica gel with a CH_2_Cl_2_-CH_3_OH gradient (*v*/*v* 100:0~0:100) to give five subfractions (Fr.2-1–Fr.2-5). Filtration of the obtained Fr.2-5 (0.68 g) over Sephadex LH-20 eluted with CHCl_3_-CH_3_OH (*v*/*v* 1:1) followed by ODS column chromatography and HPLC with a Hypersil ODS2 column (5 μm, 4.6 × 250 mm) and CH_3_OH:H_2_O in a ratio of 63:37 (*v*/*v*, 4 mL/min) provided compounds **3** (15 mg, t_R_ = 97 min), **4** (21 mg, t_R_ = 79 min), and **5** (3 mg, t_R_ = 62 min). Fr.3 (0.48 g) was subjected to a Sephadex LH-20 column eluted with CHCl_3_-CH_3_OH (*v*/*v* 1:1) followed by ODS column chromatography to give Fr.3-3-5. Fr.3-3-5 was further purified by HPLC using a Sinochrom ODS-AP column (5 μm, 4.6 × 200 mm) and MeOH:H_2_O in a ratio of 54:46 (*v*/*v*, 0.95 mL/min) to yield compounds **1** (3 mg, t_R_ = 130 min) and **2** (11 mg, t_R_ = 87 min). Fr.5 (0.43 g) was also fractionated through Sephadex LH-20 gel filtration chromatography eluted with CHCl_3_-CH_3_OH (*v*/*v* 1:1). The obtained Fr.5-3 (0.195 g) was purified by ODS column chromatography followed by HPLC with a Sinochrom ODS-AP column (5 μm, 4.6 × 200 mm) and MeOH:H_2_O in a ratio of 55:45 (*v*/*v*, 0.95 mL/min) to give compound **6** (3.5 mg, t_R_ = 65 min).

2H-(2*E*)-tricycloalternarene 12a (1): colorless oily solid (CHCl_3_); UV (MeOH) λ_max_ (log*ε*) 263 (4.23) nm; CD (MeOH) λ_max_ (Δ*ε*) 208 (−0.36) nm, 229 (1.32) nm, 267 (−0.61) nm, 306 (0.59) nm; ^1^H- and ^13^C-NMR is given in Table 1. HR-ESI-MS *m*/*z* 391.2497 [M + H]^+^, 413.2316 [M + Na]^+^ and 803.4724 [2M + Na]^+^ (calcd. for C_23_H_34_O_5_Na 413.2304).

### 3.4. Cytotoxicity Assay

The in vitro cytotoxicity of tricycloalternarene compounds was evaluated using the MTT method. Tumor cells were seeded in 96-well plates at a density of 1 × 10^4^ cells/well in 100 μL RPMI 1640 medium, and the subsequent incubation was permitted at 37 °C with 5% CO_2_ for 24 h before assessment. Compounds at preset concentrations were added to five replicate wells and treated cells for 48 h, respectively, with cisplatin as a positive reference. MTT (10 μL) was added to each well and the plates were continued to culture for 4 h. Then, the supernatant of each well was removed and 150 μL DMSO was added to dissolve the formazan. The absorbance at 490 nm of each well was measured using an automatic ELISA plate reader (ELX800UV, BioTek Instruments, Inc., Winooski, VT, USA). The experiments were repeated three times. Cell viability was expressed as percentage of cell proliferation in comparison to the negative control.

### 3.5. Cell Cycle Analysis

SMMC-7721 cells were seeded in 6-well plates at 1 × 10^6^ cells/well, and treated with compounds at different concentrations at 37 °C with 5% CO_2_ for 48 h. The trypsin-digested cells were collected and centrifuged at 1000 r/min for 5 min, then fixed in 70% cold ethanol at 4 °C for at least 18 h. They were then centrifuged at 2400 r/min for 5 min, re-suspended in 0.4 mL PI solution (containing 50 μg/mL PI and 50 μg/mL RNaseA), and incubated at 37 °C for 30 min. Finally, they were filtrated through 400 mesh screening and subjected to FACSCalibur Flow Cytometry. Cell cycle analysis was performed using ModFit LT3.0 (Becton, Dickinson and Company, Franklin Lakes, NJ, USA).

### 3.6. Detection of Apoptosis

Annexin V-FITC/PI dual stain assay was used to detect the apoptosis of SMMC-7721 cells. Tumor cells were seeded in 6-well plates at 1 × 10^6^ cells/well, and treated with compounds at different concentrations. After incubation for 24 h, cells were washed with cold PBS and re-suspended in 100 μL Annexin V/PI binding buffer and incubated with 5 μL of Annexin V and 1 μL of PI for 15 min at room temperature in the dark. Viable cells were scored as those that were negative for Annexin V and PI. The stained cells were analyzed by flow cytometry to determine the percentages of cells who had undergone apoptosis, including Annexin V^+^/PI^−^ (early apoptosis) and Annexin V^+^/PI^+^ (late apoptosis) cells.

### 3.7. Western Blot Analysis

Equal proteins were resolved by SDS-PAGE and transferred onto PVDF membranes. The membranes were blocked in 5% nonfat dry milk in TBST (TBS-0.05% Tween-20) with gentle shaking for 2 h, and then incubated with primary antibody overnight at 4 °C. After washing with TBST three times, the membranes were incubated with the appropriate HRP-conjugated secondary antibody, and then detected using the Super ECL Plus ultra-sensitive liquid. Images were collected and the respective bands were quantitated by densitometric analysis using the Image J program.

### 3.8. Statistical Analysis

Data were expressed as means ± SD. IC_50_ was calculated through improved karber’s method. Figures were obtained using Graphpad 5.0 and the difference between two groups was analyzed by *t*-test in Excel 2013. * *p* < 0.05 indicated a significant difference, while ** *p* < 0.01 indicated an even greater significant difference.

## 4. Conclusions

A new tricycloalternarene compound, 2H-(2*E*)-tricycloalternarene 12a (**1**), along with five known analogs (**2**–**6**), was isolated from the liquid culture of the endophyte *Alternaria* sp. W-1 associated with *L. japonica*. Compounds **1**, **3**, and **4** showed moderate cytotoxicity against SMMC-7721 cells and compounds **3** and **6** inhibited the growth of SGC-7901 cell lines to some extent. Further studies revealed that the in vitro anticancer activity of compound **3** against SMMC-7721 cells was related to induced G1 phase cell cycle arrest and induced apoptosis. Moreover, the induced apoptosis was caspase-dependent and involved in both the mitochondrial pathway and the death receptor pathway. This is the first report on the anticancer mechanism of tricycloalternarene compounds.

## Figures and Tables

**Table 1:** ^1^H- (600 MHz) and ^13^C-NMR (150 MHz) data of 2H-(2*E*)-tricycloalternarene 12a (**1**) in DMSO-*d*_6_.

No.	*δ*_C_	*δ*_H_, Mult. (*J* in Hz)
1	68.4	3.83, dd (10.8, 6.0); 3.75, dd (10.8, 6.6)
2	31.7	1.64, m
2′	16.5	0.81, d (7.2)
3	32.6	1.23, m; 1.00, m
4	23.9	1.15 *; 1.15 *
5	34.4	1.36, m; 1.15 *
6	31.9	1.89 *
6′	20.1	0.90, d (6.6)
7	149.5	-
8	119.7	5.31, s
9	44.3	2.52, m; 2.43 *
10	87.3	-
10′	23.0	1.40, s
11	46.0	2.76, br d (4.8)
12	14.8	2.43 *; 2.09, m
13	106.7	-
14	170.8	-
15	64.6	4.01, t (4.8)
16	29.6	1.91 *; 1.80, m
17	32.2	2.47, m; 2.08, m
18	195.7	-
1′	170.3	-
1″	20.6	2.00, s
15-OH	-	5.38, s

* Overlapped multiplet.

## Supplementary Materials

The following are available online at http://www.mdpi.com/1660-3397/16/11/402/s1: Figure S1: ^1^H-NMR spectrum of **1** (600 MHz, DMSO-*d*_6_), Figure S2: ^13^C-NMR spectrum of **1** (150 MHz, DMSO-*d*_6_), Figure S3: HSQC spectrum of **1** (600 MHz, DMSO-*d*_6_), Figure S4: HMBC spectrum of **1** (600 MHz, DMSO-*d*_6_), Figure S5: ^1^H-^1^H COSY spectrum of **1** (600 MHz, DMSO-*d*_6_), Figure S6: NOESY spectrum of **1** (600 MHz, DMSO-*d*_6_), Figure S7: CD spectra of **1** and **2**.
