# Supplementary material for: Cytotoxic Tricycloalternarene Compounds from Endophyte Alternaria sp. W-1 Associated with Laminaria japonica
Source: Mar Drugs. 2018 Oct 23;16(11):402. doi: 10.3390/md16110402 (PMC6267107; doi:10.3390/md16110402)
Supplement: Supplementary file 1 [file marinedrugs-16-00402-s001.pdf]

marine drugs

Article

Cytotoxic tricycloalternarene compounds from endophyte *Alternaria* sp. W-1 associated with *Laminaria japonica*

Li Shen[1,2,3], Shu-Juan Tian[4], Hui-Liang Song[1], Xi Chen[1], Hao Guo[1], Dan Wan[1], Yu-Rou Wang[1], Feng-Wu Wang[4,*] and Li-Jun Liu[5,*]

1 Institute of Translational Medicine, Medical College, Yangzhou University, Yangzhou 225001, China; shenli@yzu.edu.cn (L.S.); 18362825104@163.com (H.-L.S.); cx0811chenxi@163.com (X.C.); 18752789443@163.com (H.G.); 18252717215@163.com (D.W.); xiaorouw@gmail.com (Y.-R.W.)

2 Jiangsu Key Laboratory of Integrated Traditional Chinese and Western Medicine for Prevention and Treatment of Senile Diseases, Yangzhou University, Yangzhou 225001, China

3 Jiangsu Co-innovation Center for Prevention and Control of Important Animal Infectious Diseases and Zoonoses, Yangzhou 225009, China

4 College of Food Science and Engineering, Qingdao Agricultural University, Qingdao 266109, China; tianshujuan1992@163.com (S.-J.T.)

5 Jiangsu Co-Innovation Center for Modern Production Technology of Grain Crops/Jiangsu Key Laboratory of Crop Genetics and Physiology, Yangzhou University, Yangzhou 225009, China

* Correspondence: wangfengwude@126.com (F.-W.W.); ljliu@yzu.edu.cn (L.-J.L.); Tel./Fax: +86-532-88030473 (F.-W.W.); ; +86-514-8797-2133 (L.-J.L.)

Content

Figure S1. ^{1}H-NMR spectrum of **1** (600 MHz, DMSO-d_6).
Figure S2. ^{13}C-NMR spectrum of **1** (150 MHz, DMSO-d_6).
Figure S3. HSQC spectrum of **1** (600 MHz, DMSO-d_6).
Figure S4. HMBC spectrum of **1** (600 MHz, DMSO-d_6).
Figure S5. ^{1}H-^{1}H COSY spectrum of **1** (600 MHz, DMSO-d_6).
Figure S6. NOESY spectrum of **1** (600 MHz, DMSO-d_6).
Figure S7. CD spectra of **1** and **2**.

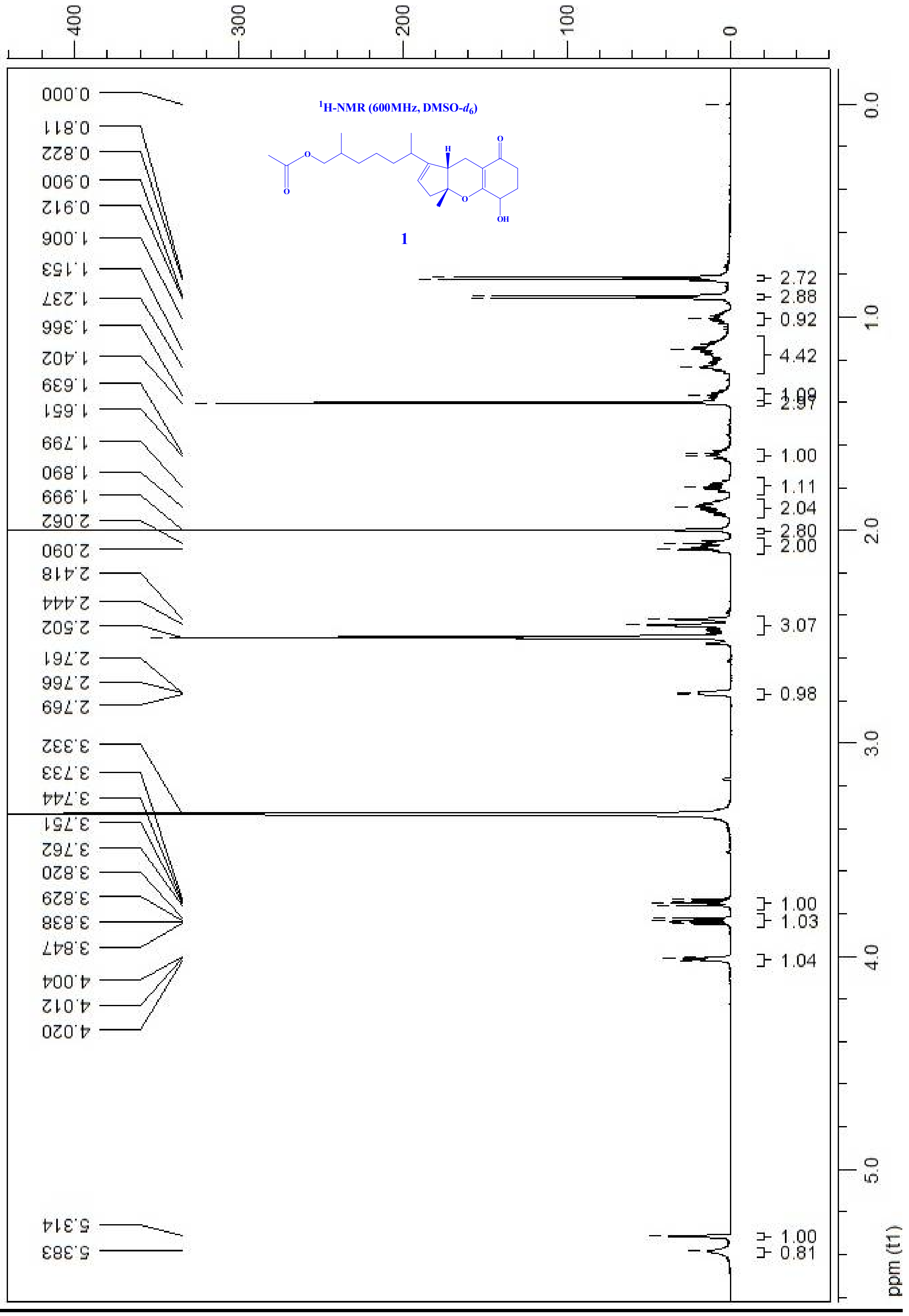

Figure S1. ^{1}H-NMR spectrum of **1** (600 MHz, DMSO-d_6).

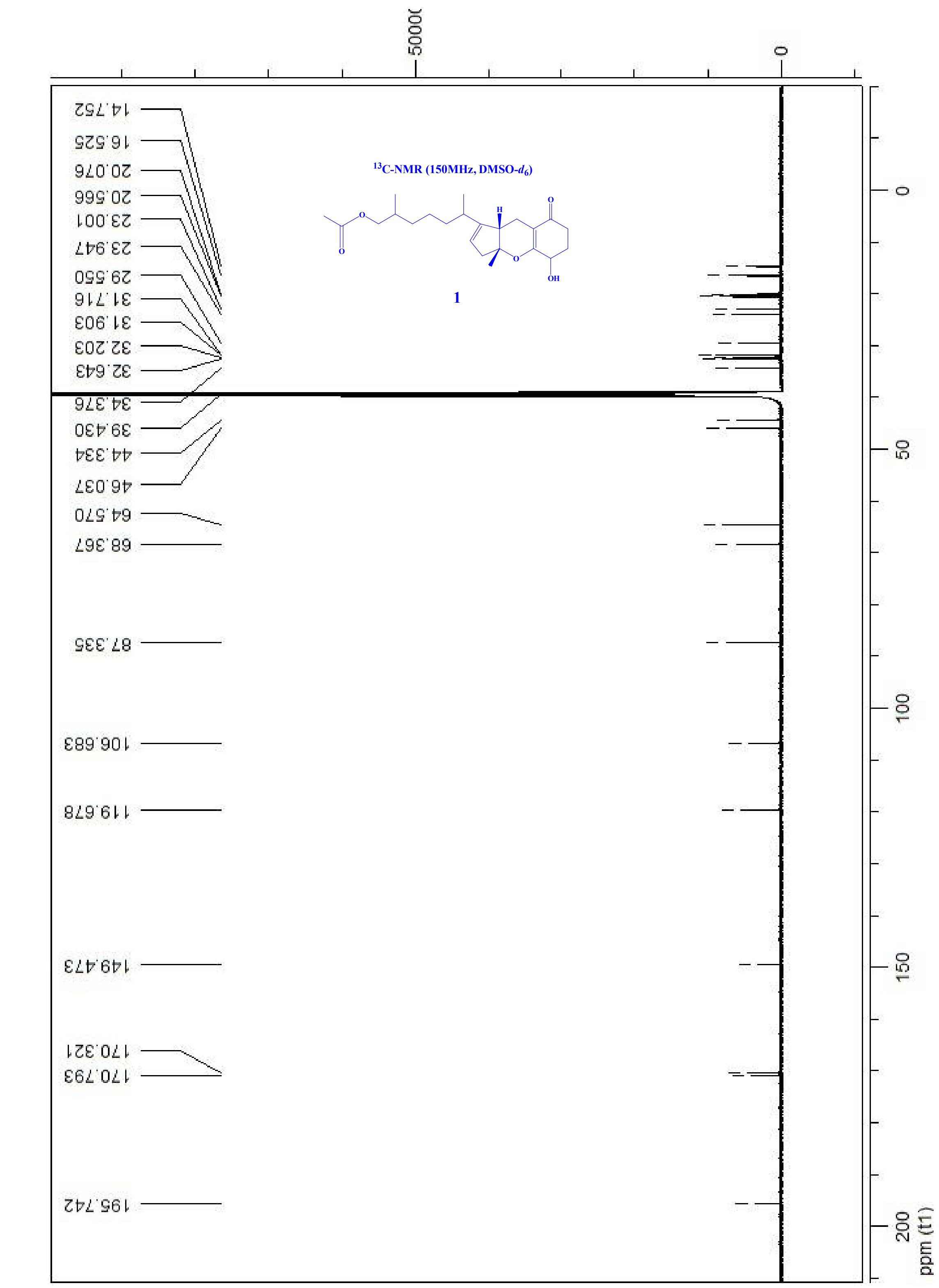

Figure S2. ^{13}C-NMR spectrum of **1** (150 MHz, DMSO-*d*$_6$).

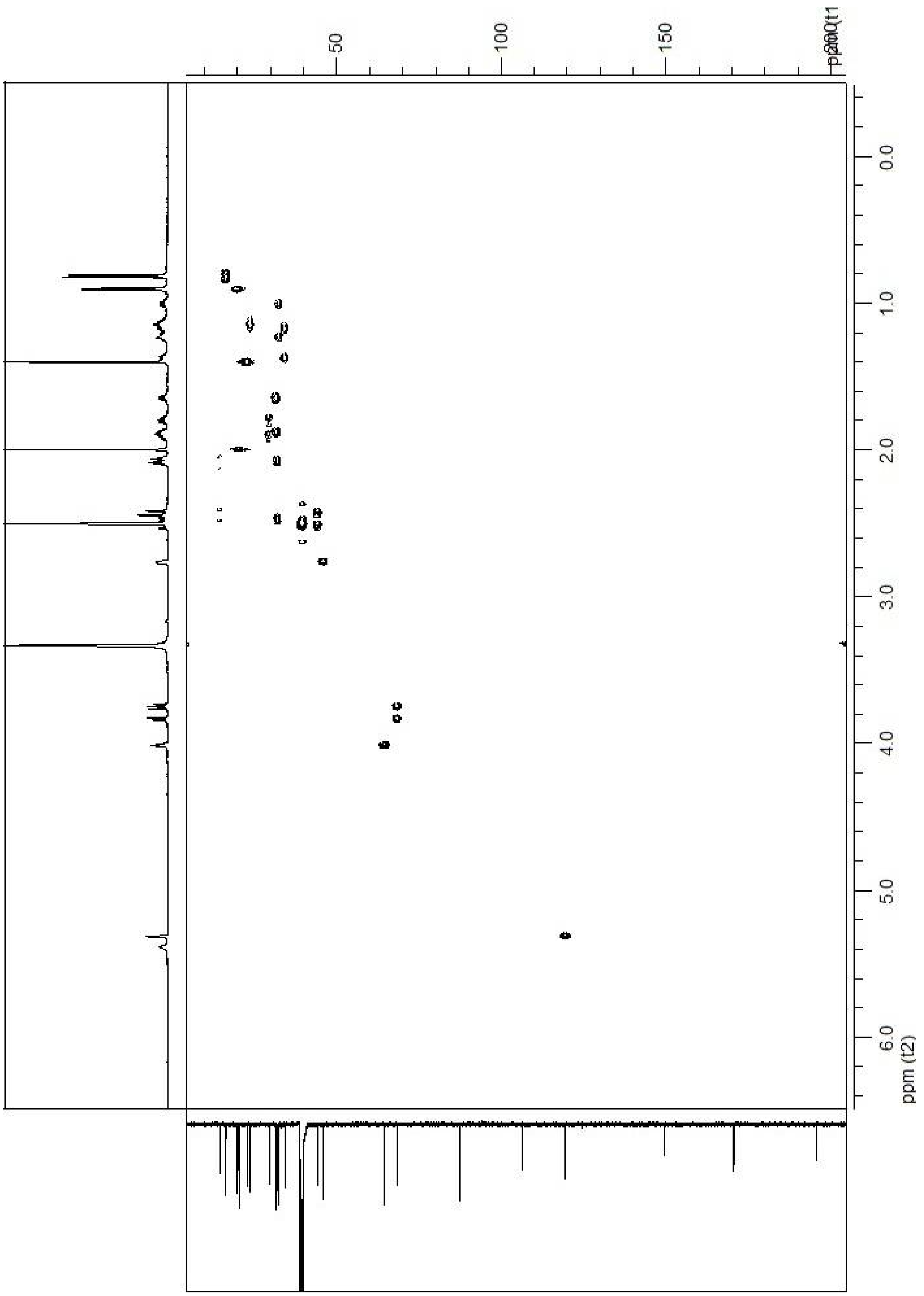

Figure S3. HSQC spectrum of **1** (600 MHz, DMSO-d_6).

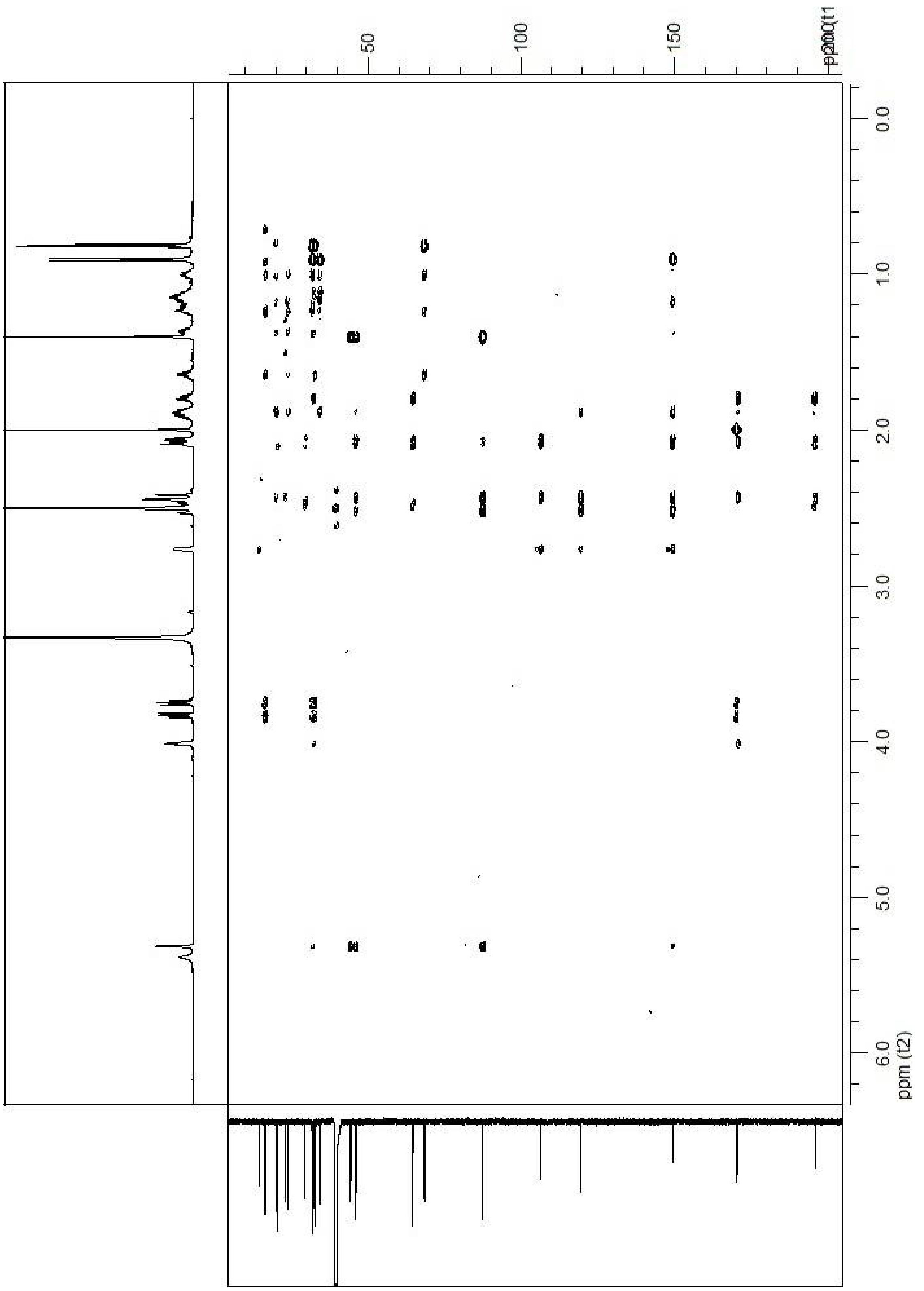

Figure S4. HMBC spectrum of **1** (600 MHz, DMSO-d_6).

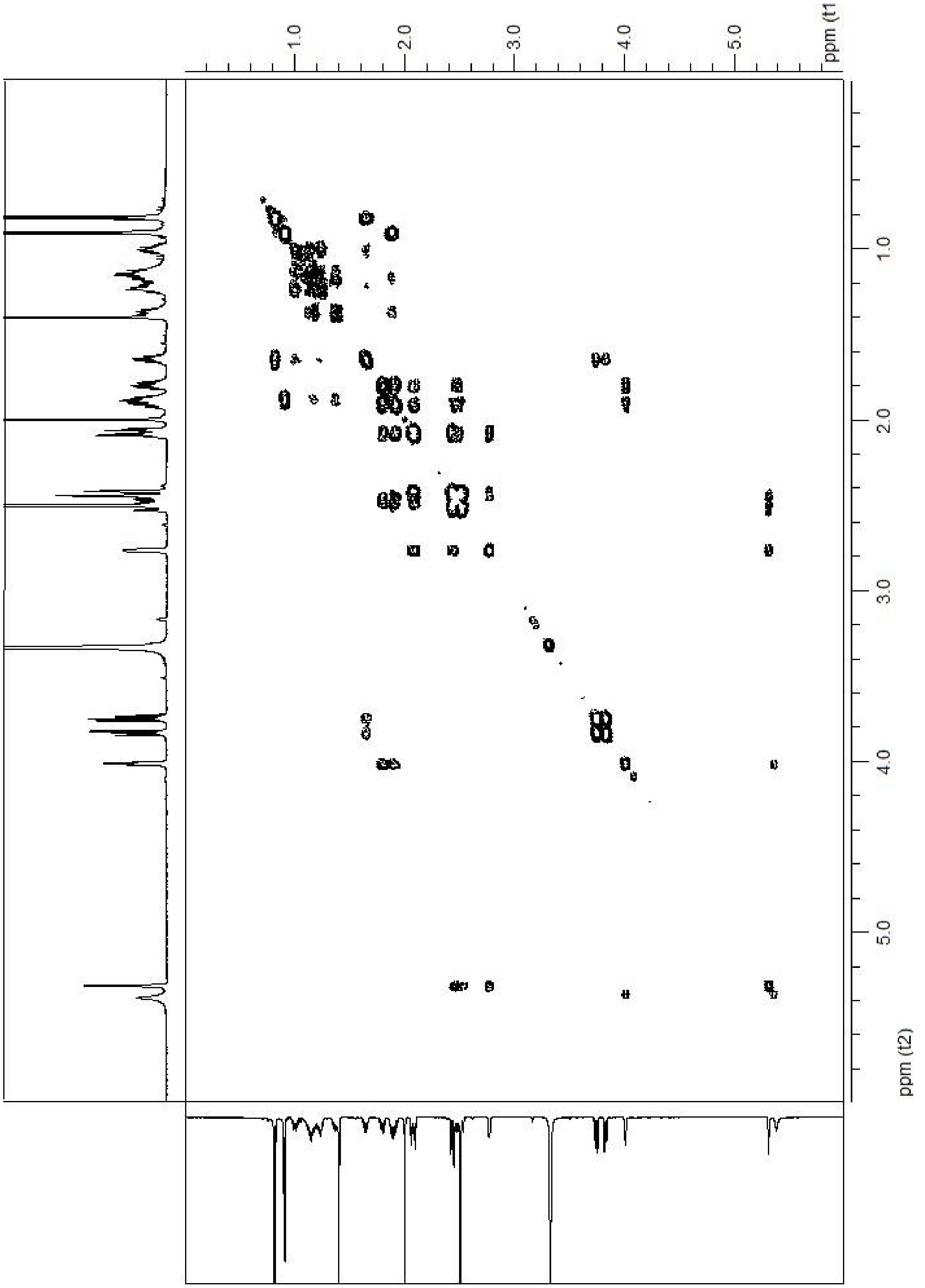

Figure S5. ^{1}H-^{1}H COSY spectrum of **1** (600 MHz, DMSO-*d*$_6$).

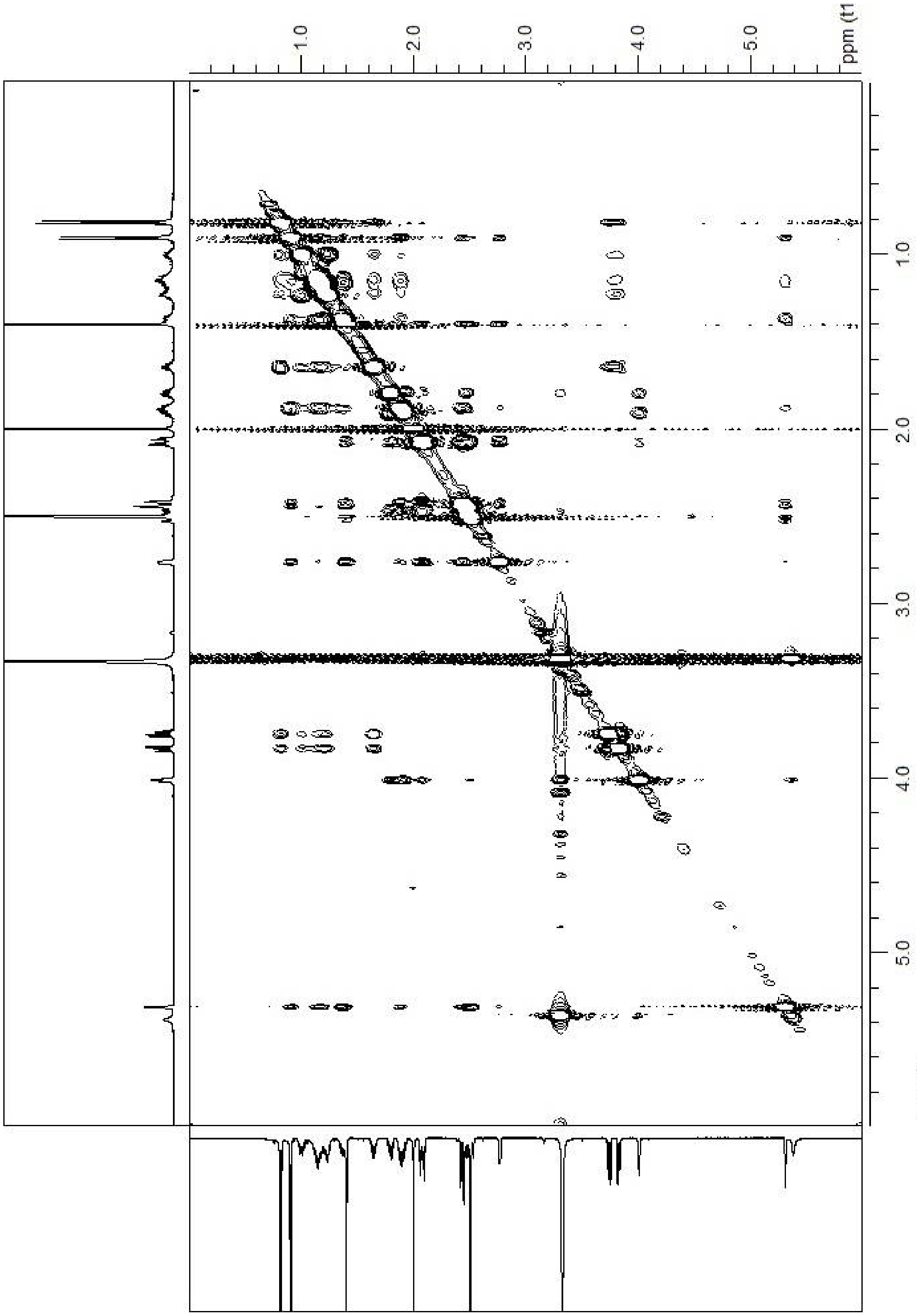

Figure S6. NOESY spectrum of **1** (600 MHz, DMSO-d_6).

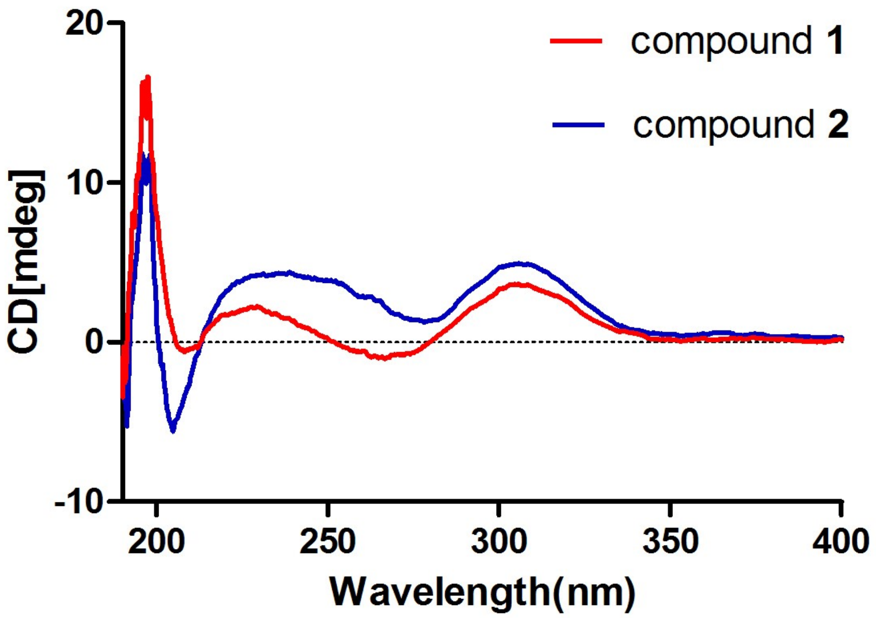

Figure S7. CD spectra of **1** and **2**.
